# Iatrogenic Ascending Aorta Dissection during Diagnostic Coronary Angiography: Rare but Life-Threatening

**DOI:** 10.1155/2014/809398

**Published:** 2014-06-16

**Authors:** Marc Lambelin, Luc Janssens, Luc Haenen

**Affiliations:** ^1^Department of Cardiology, Imelda Hospital, Imeldalaan 9, 2820 Bonheiden, Belgium; ^2^Department of Cardiology, University Hospital Leuven, Herestraat 49, 3000 Leuven, Belgium; ^3^Department of Cardiovascular Surgery, Imelda Hospital, Imeldalaan 9, 2820 Bonheiden, Belgium

## Abstract

Dissection of the ascending aorta is a very rare but life-threatening complication during diagnostic angiography. We present a case of an elderly woman who underwent an elective diagnostic coronary angiography, complicated with an iatrogenic ascending aorta dissection that did not involve the coronary arteries but originated 4 cm distal of the aortic valve. The patient developed cardiogenic shock due to acute pericardial tamponade and so immediate, life-saving cardiac surgery with implantation of a supracoronary graft was successfully performed. A biopsy from the excised aorta showed loss of smooth muscle cells and accumulation of basophilic ground substance, clear features of cystic media necrosis. This is believed to be the underlying cause of the dissection besides a nonselective injection of the right coronary artery.

## 1. Introduction

Besides puncture-related minor complications, diagnostic coronary angiography is a frequently performed procedure with a very low risk, especially when performed in an elective situation (<1.3%) [1]. Emergency coronary angiogram or percutaneous coronary interventions carry a much higher complication risk.

We report the case of an elderly woman who developed a dissection of the ascending aorta without coronary involvement during diagnostic angiography. The dissection was complicated by pericardial tamponade and emergency surgery with implantation of a supracoronary graft was successfully performed.

Risk factors for developing such aortic dissection are underlying pathologic changes of the aortic wall, a nonselective contrast injection, and the use of abnormal catheters [2].

## 2. Case Report

A 75-year-old woman was admitted for elective coronary angiography with right heart catheterization because of a severe aortic regurgitation grade 4/4 and dyspnea NYHA III. In her medical history, we report hypothyroidism, arterial hypertension, and dyslipidemia. Her familial history was unremarkable. Coronary angiogram revealed no coronary lesions. The left ventricular function (LVEF) was 69% without evidence of any regional wall abnormality. Filling pressures were normal (PCWP 16 mmHg, PAP 33/18/23 mmHg).

Right side pressures were measured via the right femoral vein and then the coronary angiography was performed via the right radial artery using a 5 Fr arterial sheath. After introducing the arterial sheath we injected 5000 IU of heparin and 1 mg of nicardipine in the radial artery.

The coronary angiogram of the right coronary artery (RCA) was performed with a 5 Fr Radial catheter (Kimal, Inc.; UK), followed by a left coronary angiogram with a 5 Fr JL 4.0 (Cordis, Cordis Corporation; USA). For the ventricular angiogram, a pigtail PIG-145° (Cordis, Cordis Corporation; USA) was used.

**Figure 1:**
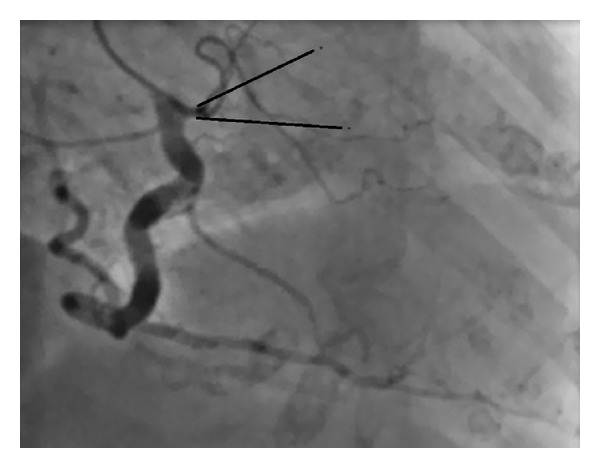
RCA in RAO. Nonselective engagement of the ostium of the RCA.

**Figure 2:**
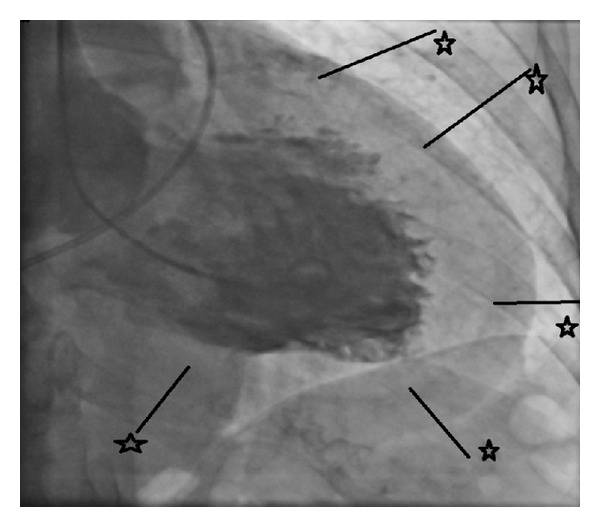
Pericardial tamponade with clear distinction of epicardial border (⋆) and pericardium.

After the first conventional injection of the RCA, the following injection was made in a not perfectly coaxial engagement of the ostium of the RCA and so it was not a selective one (Figure 1). It passed without any pain or discomfort. Because of the enlargement of the ascending aorta, the JL 4.0 catheter was placed in a rather unusual position for cannulating the LCA. Four angiograms of the LCA were made with a steady, adequate position of the JL catheter (Figure 2).

**Figure 3:**
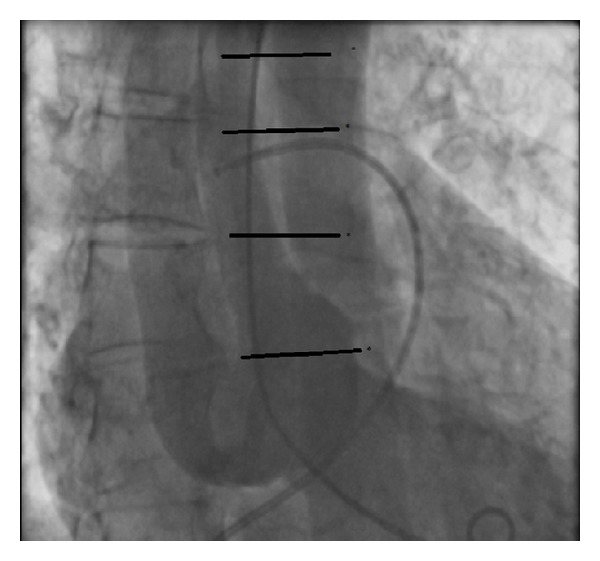
Dissection flap in the ascending aorta.

After the last injection of the LCA, the patient suddenly complained of a heavy chest and back pain, irradiating to the shoulders with an instant drop in blood pressure till SBP 50 mmHg. During the ventricular angiogram, a huge, contrast-negative mobile linear image was recognized, highly suggestive of an aortic intimal tear (Figure 3). Meanwhile, the patient developed a cardiogenic shock because of cardiac tamponade. The patient was immediately transferred to the operating room. (See Supplementary Material available online at http://dx.doi.org/10.1155/2014/809398).

A transesophageal echocardiography (TOE) showed a tricuspid aortic valve and an aortic annulus that was in the upper limit of normal (24 mm, 13.8 mm/m²). The ascending aorta was mildly dilated (38 mm, 21.8 mm/m²) as well as the sinuses of Valsalva (45 mm, 25.8 mm/m²) and the sinotubular junction (38 mm, 21.8 m/m²).

A supracoronary replacement of the ascending aorta was performed. After implantation of the supracoronary graft, TOE demonstrated a sufficient tricuspid aortic valve that could be left in situ.

During angiography the entry site of the dissection could not be established, but during the operation, the surgeon could point out the intimal tear at 4 cm distal of the aortic valve. Thus, the coronary arteries had no need for surgical intervention.

Further postoperative course was uneventful. A transthoracic echocardiography in the outpatient clinic more than 2 months after aortic surgery could not demonstrate any residual aortic regurgitation.

## 3. Discussion

Coronary dissection extending retrograde towards the sinus of Valsalva and the ascending aorta during percutaneous interventional procedures is a well-recognized complication (0.01–0.07%), but a dissection of the ascending aorta during diagnostic angiography is very rare (0.001–0.02%) [2–4]. A diagnostic procedure in an emergency setting, such as acute myocardial infarction, carries a much higher risk than one performed in elective settings (0.1% in AMI, <0.01% in elective settings) [5].

**Figure 4:**
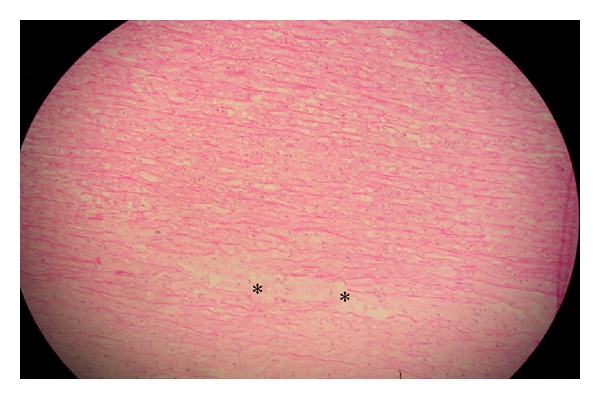
Accumulation of basophilic ground substance (∗) in cystic media degeneration and necrosis.

In a biopsy, taken from the resected aortic wall, intrinsic abnormalities of the aortic wall could be demonstrated by loss of smooth muscle cells and accumulation of basophilic ground substance, defining cystic media degeneration and necrosis (CMD) (Figure 4). Several surgical pathology series point out CMD as the most common histologic finding in aortic dilatation and dissection with an increasing severity of CMD in older patients [6–8]. In iatrogenic aortic dissection, atherosclerosis tend to be the most important histologic finding (61.2%) before CMD (22.2%) [3].

The exact mechanism responsible for the occurrence of aortic dissection during diagnostic angiography remains to be established. In this case, the nonselective contrast injection is believed to play a major role with underlying CMD. An unusual coronary engagement and the use of nonconventional catheters are also often involved in causing aortic dissection during diagnostic angiography [2].

If an aortic dissection occurs as a retrograde propagation of a coronary dissection, the therapy of choice is sealing the entry site with a stent implantation followed by a conservative management and transesophageal echography controls. However, if the dissection is extending more than 4 cm above the coronary sinuses, it usually requires surgical management [9].

In this extremely rare case, the entry site was not a coronary dissection, but it was located 4 cm distal of the aortic valve. Furthermore, the patient was developing cardiogenic shock so that immediate surgical repair was imperative. After implantation of the graft from the supracoronary region unto the origin of the brachiocephalic trunk and thus reducing the diameter of the aorta and sinotubular junction, there was a perfect coaptation of the aortic valve cusps. So the aortic valve could be left in situ. This kind of valve sparing operation can be performed with an excellent long-term result with 87% freedom of aortic regurgitation grade II or more after 10 years and <10% reoperation [10, 11].

In summary, this case demonstrates an iatrogenic acute aortic dissection with a supracoronary entry without involvement of the coronary arteries which occurred during diagnostic coronary angiography. It most likely resulted from the combination of underlying cystic media necrosis, a mildly dilated aorta, and a nonselective contrast injection in the right coronary artery. Fortunately, immediate and appropriate measures were undertaken and the patient recovered well after replacement of the supracoronary ascending aorta.

## Supplementary Material

Sudden onset of heavy chest and back pain during ventricular angiogram, coinciding an instant drop in blood pressure due to pericardial tamponade. The tamponade is the result of type A dissection, originating from an intimal tear in the ascending aorta.
